# How to select a best-value biological medicine? A practical model to support hospital pharmacists

**DOI:** 10.1093/ajhp/zxac235

**Published:** 2022-08-25

**Authors:** Liese Barbier, Yannick Vandenplas, Niels Boone, Isabelle Huys, Rob Janknegt, Arnold G Vulto

**Affiliations:** Department of Pharmaceutical and Pharmacological Sciences, KU Leuven, Leuven, Belgium; Department of Pharmaceutical and Pharmacological Sciences, KU Leuven, Leuven, Belgium; Hospital Pharmacy, Zuyderland Medical Center, Heerlen, the Netherlands; Department of Pharmaceutical and Pharmacological Sciences, KU Leuven, Leuven, Belgium; Sittard-Geleen, the Netherlands; Department of Pharmaceutical and Pharmacological Sciences, KU Leuven, Leuven, Belgium; Hospital Pharmacy, Erasmus University Medical Center, Rotterdam, the Netherlands

**Keywords:** best-value biological, biological, biosimilar, procurement, selection

## Abstract

**Purpose:**

With the growing availability of biosimilars on the global market, clinicians and pharmacists have multiple off-patent biological products to choose from. Besides the competitiveness of the product’s price, other criteria should be considered when selecting a best-value biological. This article aims to provide a model to facilitate transparent best-value biological selection in the off-patent biological medicines segment.

**Summary:**

The presented model was developed on the basis of established multicriteria decision analysis tools for rational and transparent medicine selection, ie, the System of Objectified Judgement Analysis and InforMatrix. Criteria for the model were informed by earlier research, a literature search, and evaluation by the authors. The developed model includes up-to-date guidance on criteria that can be considered in selection and provides background on the allocation of weights that may aid hospital pharmacists and clinicians with decision-making in practice. Three main categories of criteria besides price were identified and included in the model: (1) product-driven criteria, (2) service-driven criteria, and (3) patient-driven criteria. Product-driven criteria include technical product features and licensed therapeutic indications. Service-driven criteria consist of supply conditions, value-added services, and environment and sustainability criteria. Patient-driven criteria contain product administration elements such as ease of use and service elements such as patient support programs. Relative weighting of the criteria is largely context dependent and should in a given setting be determined at the beginning of the process.

**Conclusion:**

The practical model described here may support hospital pharmacists and clinicians with transparent and evidence-based best-value biological selection in clinical practice.

**KEY POINTS**

- Hospital pharmacists experience difficulties with formulating and applying criteria besides price in the context of selection of off-patent biologicals and biosimilars, highlighting the need for guidance.
- This article provides an up-to-date and transparent model, which attempts to guide hospital pharmacists regarding possible criteria to consider in the selection of a best-value biological, or best-value biologicals, in clinical practice.
- Possible criteria to consider, besides price, when selecting a best-value biological can be categorized into 3 groups: product-driven, service-driven, and patient-driven criteria.

Since approval of the first biosimilar in Europe in 2006, more than 70 biosimilars across multiple therapeutic areas have been licensed and considerable experience has been gathered with biosimilar use in clinical practice.^1^ In the US, the first biosimilar received regulatory approval in 2015. Since then, over 30 biosimilar products have been licensed by the Food and Drug Administration (FDA).^2^ Despite an initial hesitancy from stakeholders to use them, biosimilars are an integrated part of clinical care in many regions today. The number of approved biosimilars is expected to grow substantially, with twice as many originator biologicals losing protection in the next 10 years.^3^

The remit and responsibilities of hospital pharmacists may vary between regions and healthcare systems,^4^ but in general they take the lead in clinical, economic, and practical considerations related to pharmaceuticals and their introduction in the hospital therapeutic formulary.^5^ Effective and well-thought-out product selection is crucial to ensure the availability of safe, effective, high-quality, and cost-effective medicines.^5^ Hospital pharmacists have the expertise to integrate criteria in product selection beyond the product’s price,^5^ allowing selection based on the broader value of the product, in other words, the selection of a best-value medicine.

Selection of a best-value biological, which can be either the originator biological or its biosimilar(s), that considers criteria beyond price in the decision-making process is a challenging and evolving topic. In response to the market entry of biosimilars, several articles have been published in the *European Journal of Hospital Pharmacy* with the aim to offer guidance on how to select a biosimilar in clinical practice.^6-8^ The biosimilar landscape has progressed considerably since these papers were published in 2005, 2008, and 2013,^6-8^ which asks for a reassessment and continued development of guidance in this regard. First, insights into the evaluation of biosimilars and their use in practice have been consolidated, making the need for certain previously proposed criteria obsolete. For example, earlier publications suggested evaluation of elements related to the biosimilar’s efficacy and safety. However, the robust European and US regulatory frameworks for the evaluation of biosimilars and the evidence acquired over 15 years of clinical experience with biosimilars have clearly demonstrated that there is no need to reassess elements that are part of regulatory evaluation once a biosimilar is licensed.^9-11^ Second, selecting a best-value biological has evolved from making a choice between a reference product and biosimilar to a choice between a reference product and multiple biosimilars and/or between biosimilars, as for almost all reference products multiple biosimilars are available on the market today. Third, companies have increasingly made efforts to differentiate their products (both originator and biosimilars) on the basis of value-adding criteria, instead of focusing exclusively on competition on price.^12^ Fourth and finally, a recent research study on biosimilar tender practices in the European Union (EU) found that purchasers, including hospital pharmacists, experience difficulties with identifying criteria besides price to use when selecting between available off-patent biologicals and biosimilars with their appropriate formulation.^13^

In this article, we provide an up-to-date model to aid hospital pharmacists and clinicians with best-value biological selection in the off-patent context, including guidance on criteria that can be considered and background on the allocation of weights and scoring.

## Literature review

This study presents a model for the selection of best-value biologicals in the off-patent context based on the System of Objectified Judgement Analysis (SOJA) and InforMatrix, 2 established assessment tools in rational and transparent drug decision-making.^14,15^ SOJA and InforMatrix are examples of multicriteria decision analysis (MCDA) tools. MCDA is defined as “a set of methods and approaches to aid decision-making, where decisions are based on more than one criterion, which makes explicit the impact on the decision of all the criteria applied and the relative importance attached to them.” ^16^

**Figure 1.**
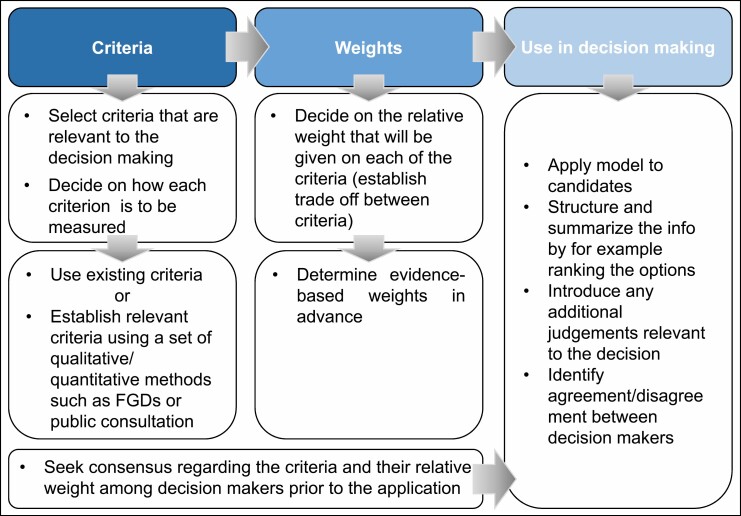
Schematic overview of the decision-making model. Figure based on and adapted from reference ^15^. FGDs indicates focus group discussions.

The decision-making model presented includes 3 consecutive steps: (1) identifying the criteria to apply in decision-making and deciding on (2) their relative weights and (3) a scoring system evaluating the possible candidates. Figure 1 provides a schematic overview of the model.

The authors identified and assessed different criteria for selecting a best-value biological medicine on their eligibility. Selection criteria can be determined by making use of existing criteria or by establishing de novo relevant criteria. For the latter approach, qualitative and/or quantitative methods, such as, for example, focus group discussions or a public consultation, have been applied in the past (Table 1).^16^ To inform this model, hand searches of the published scientific literature in PubMed, Embase, and gray literature were performed. Search terms were related to biosimilars, biologicals, procurement, and tendering. No formal inclusion or exclusion criteria were established. Identified articles were reviewed qualitatively by the researchers. The criteria identified were compiled, compared, and evaluated on the basis of the SOJA and InforMatrix model criteria and discussion among the authors. Specifically, criteria were assessed for their compatibility with the biosimilarity principle and their relevance today.

**Table 1.** Overview of Publications in Which Criteria to Evaluate and/or Select Off-patent Biologicals and Biosimilars Were Generated

Study	Year	Information on criteria	Methodology
Crommelin et al^6^	2005	Development of checklist to evaluate biosimilars	Preparation by an international working group, involving scientists, hospital pharmacists, and representatives from a manufacturing company (based on an advisory board meeting)
Kramer et al^7^	2008	Development of a checklist to guide originator and biosimilar evaluation	Further development of 2005 checklist by authors
Boone et al^8^	2013	Development of a shortlist of criteria to guide biosimilar selection	Identification of criteria by involved researchers and evaluation of criteria with SOJA and InforMatrix tools
Griffitth et al^17^	2014	Development of formulary selection criteria for biosimilars (US focus)	No information on methodology
Barbier et al^13^	2021	Overview of possible criteria to consider and steer away from when selecting a best-value biological	Overview of criteria informed by quantitative (by means of a web survey across EU countries) and qualitative (by means of semistructured interviews with EU experts) insights from suppliers and purchasers

Abbreviations: EU, European Union; SOJA, System of Objectified Judgement Analysis.

On the basis of this, we present an up-to-date overview of possible criteria to consider when selecting best-value biological(s). Furthermore, we provide the necessary context that may assist hospital pharmacists and clinicians when choosing criteria appropriate to the product and their decision-making context.

## Criteria to select a best-value biological

Criteria for selection should allow an objective comparative assessment of the multiple candidates. Criteria need to be transparently formulated, factually measurable, and differentiating without being discriminatory.^13^

**Figure 2.**
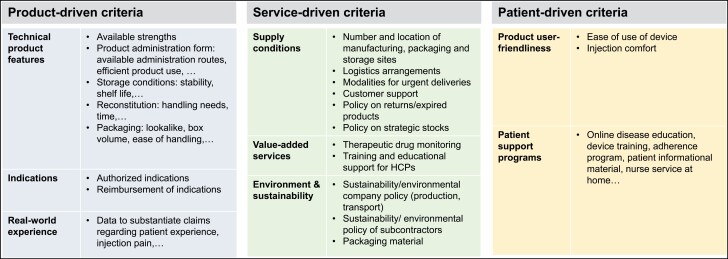
Overview of possible criteria to consider, besides price, when selecting a best-value biological. HCPs indicates healthcare professionals.

An overview of possible criteria that can be considered for best-value biological selection is given in Figure 2. The criteria are classified into 3 main categories: product-driven, service-driven, and patient-driven criteria.

## Product-driven criteria

### Technical product features.

Differentiation on product-related elements such as presentation (including available strengths and administration routes), reconstitution, storage conditions, and packaging may provide products with a competitive advantage over their alternatives. eTable 1 provides an example of differences in presentation for a selection of biologicals (reference product and biosimilars).

### Available strengths.

The availability of multiple, and especially more, strengths compared to other candidates can have both economic and operational advantages. Multidose vials may allow clinicians to better tailor to the dosage needs of individual patients, resulting in a more efficient use of resources (less spillage).^18^ The higher the number of strengths available, the higher the product could be scored on this criterion.

### Product administration.

Product availability in multiple formulations can provide products with a competitive advantage as this offers multiple treatment options (for the patient and the healthcare provider) to choose between. For instance, trastuzumab and rituximab, both originally approved as intravenous (IV) formulations, have also been developed for subcutaneous (SC) administration. However, not only originator developers invest in the development of alternative administration routes (eg, an SC formulation of an infliximab biosimilar, which is not available for the reference product).^19^ The SC administration route may offer advantages, by reducing in-hospital treatment time and resources compared to IV infusion. Especially hospitals with less day care capacity may benefit from a more time-efficient SC formulation. In addition, the SC administration route may be preferred by patients due to its increased convenience. This leads to the question of whether these advantages outweigh the reduced price of the IV administration form for which biosimilar competition is available.^20,21^ To adequately answer this question, product price and other cost elements (eg, IV vial sharing, healthcare professional time, hospital organization) should be included in analysis of the trade-off.^22^

Differences in infusion time (ie, demonstration of the safety of a shorter infusion time compared to other candidates), if present, could be considered when evaluating IV-administered products. Self-injectable products that offer a temperature-sensitive indicator on the injection device, showing whether the product has been stored at the appropriate temperature, may guide patients with correct medication storage. Products that require less frequent administration compared to their competitors may also receive a higher score.

For SC biologicals, the user-friendliness of the injection device and the product’s injection comfort should also be considered (see “Patient-driven criteria”).

In conclusion, availability of multiple formulations may increase patient choice and allows tailoring of formulation choice to the setup of the hospital (eg, organized to cater to IV and/or SC administration).

However, biosimilar developers cannot apply for market authorization in the US for different strengths, dosage forms, or routes of administration than are available for the reference product.^23^ As a result, products with different routes of administration are marketed as new drugs instead of biosimilars. This has already been the case for the SC infliximab CT-P13.^24^ This is in contrast to Europe, where SC infliximab CT-P13 is registered as a biosimilar.^25^

### Reconstitution.

For IV products, the product’s reconstitution should also be considered. For instance, the availability of a ready-to-use formulation could reduce the medicine handling time for healthcare providers compared to a product that requires in-hospital pharmacy preparation. The dissolution rate—relevant under everyday use conditions—could also be a point of consideration.

### Storage conditions.

Differences in storage conditions (that is, in the freezer, in the refrigerator, or at room temperature) could be scored on convenience. Cooled storage space is costly, and therefore large-volume packaging of biologicals needs to be avoided. Additionally, the product’s shelf life could be a possible differentiator. Data on extended in-use stability could be advantageous, as this may permit safe in-advance preparation, allowing optimization of pharmacy and nurse workload management.^26^ In addition, data on stability under different storage conditions (eg, storage in the refrigerator vs at room temperature) could be informative in cases where temperature deviations would occur during product transport or storage.^26^ Additional research and documentation regarding product stability can thus be included as criteria.

### Packaging.

Product packaging and labeling should be clear and easy to read. In addition, the packaging should allow sufficient differentiation with products from other suppliers and between products from the same supplier.^17^ Barcoding on the product’s per-dose packaging also aids to limit medication errors. The availability of products in per-dose packaging takes away the need for hospitals to repackage blisters to individual unit doses and may as such have a positive impact on associated pharmacy workload.

Product pack size (determining how frequently prescriptions need to be filled) should also be considered, as it may affect patient copayment in reimbursement systems that are sensitive to this.

To fight medicine falsifications and ensure safe and controlled trade, documentation regarding adherence to the EU Falsified Medicines Directive or US Drug Supply Chain and Security Act should be present. In both the EU and US, the presence of a unique identifier and antitampering device on the outer packaging of medicines is obligatory for all medicines and is thus not expected to be a differentiating element between products.^27,28^

### Therapeutic indications: authorization and reimbursement status.

Biosimilars are generally approved for the same indications as the reference product. In some instances, a biosimilar may however have fewer licensed indications than the reference product, as companies may choose not to apply for approval for all therapeutic indications of the reference product.^9^ Not all indications of the reference product may be eligible for the biosimilar to include in its label at the time of initial marketing authorization due to patent or regulatory exclusivity coverage. In addition, some licensed indications might differ between SC and IV products, such as with SC rituximab where rheumatoid arthritis is not a licensed indication.^29,30^

Although this is not yet the case for the biosimilars that are currently available in Europe, biosimilars can also obtain additional licensed indications compared to the reference product. From a regulatory point of view, it is possible for a company to apply for an additional therapeutic indication beyond the indications included in the label of the reference product for their biosimilar product upon initial marketing authorization. For this, the biosimilar applicant has to provide additional clinical data for this particular indication.^17^ Seeking additional indications during the product’s life cycle is thus a differentiating strategy that theoretically can be applied in Europe for both originators and biosimilars.

However, in the US, it is not possible for biosimilars to add extra indications compared to the reference product.^23^ As a result, in the US, biosimilar products will not be able to differentiate themselves from their reference product by seeking additional indications. Furthermore, biosimilars do not have to apply for all the registered indications of the reference product. Thus, biosimilars may have fewer authorized indications than their reference product.^23^

Additionally, the reimbursement status of the product is an important factor. In Europe, there can be differences in reimbursed indications between biological products in certain countries while the licensed indications are the same. For example, if certain indications of the original product fall under a managed entry agreement at the time of biosimilar market entry, the biosimilar company might opt out of reimbursement for this particular indication.^17^ Of note, in most European countries, there is no preferential reimbursement status for the reference biological or a biosimilar. This contrasts with the US, where healthcare payers may have preferences for a certain product. Public (ie, Medicaid, Medicare) or private health insurers may incorporate preferred coverage for a biosimilar or reference product in their medical formulary decisions.^31,32^ If a payer considers a particular biological to be the preferred treatment, this corresponds to the “fail first” principle whereby patients should be treated with this biological first. On top of this, payer preference policies can change over time, so this is another aspect to consider, especially for long-term treatments.^33^ The hospital pharmacist should therefore verify payer preferences, as treatment interruptions should be avoided at all times.

With regard to interchangeability, in the US, interchangeability is a legal status that can be obtained for a biosimilar product, as outlined in the Biologics Price Competition and Innovation Act.^34-36^ Interchangeability status is assessed and granted by FDA, after which interchangeable biologicals are allowed to be substituted at the pharmacy level without the consent of the prescriber, if permitted by state laws. Because interchangeability status primarily serves as a tool to regulate substitution across the US, it might be relevant to consider for products that are dispensed outside the hospital.

### Real-world product experience.

As biosimilar development aims to demonstrate biosimilarity to the reference product and not independently establish the efficacy and safety of the proposed product, because this is already well known for the reference product, the requirements for clinical development are different from those for the reference product. The tailored clinical development package for a biosimilar generally consists of a phase 1 study and, depending on the complexity of the product, a confirmatory efficacy and safety trial in patients in one licensed indication of the reference product.^20,37^ The clinical development package (its extensiveness, the patient setting study, etc) should however not be reassessed during best-value biological selection, as it is part of the product’s regulatory evaluation.^13^

As for any new approved medicine, utilization and clinical experience data may be informative. However, the real-world utilization of the reference product will logically outweigh that of recently approved biosimilar entrants. Biosimilar and reference products can be considered to have a similar offering, although utilization and experience may differ at the time of biosimilar market launch. In relation to switching, several national medicine agencies have provided clear guidance, indicating that no effect on efficacy or safety is to be expected when switching patients from the reference medicine to a biosimilar or vice versa.^38-40^

## Service-driven criteria

### Supply conditions.

Supply criteria are related to the pharmaceutical product’s manufacturer and may include manufacturing capacity, storage locations, modalities for urgent deliveries, customer support, policy for expired products or returns, and policy on strategic stocks. The production (including manufacturing, packaging, and storage) capacity of the company must be sufficient to guarantee supply continuity.^7,8^ Additionally, a history of possible stockouts or backorders of the supplier may be informative regarding supply reliability. Strategic stocks are useful to guarantee continuous delivery in the case of supply chain issues or batch failures.

In the context of tenders, suppliers are often selected in advance on the basis of whether they meet a certain set of requirements, including ones related to supply. Indeed, as continuous and reliable supply is of utmost importance, criteria related to supply may be a prerequisite, as minimum requirements that a supplier must meet, before applying product-specific criteria.^8^

### Value-added services (VAS).

VAS have the intention to add value to the product, in terms of improving patient and health outcomes.^41,42^ VAS are often directed at improving patient care and adherence in the hospital environment or in support of delivery of the medicine at home. They may exist in several forms or modalities, such as nurse services at home, therapeutic drug monitoring support, and training or education for healthcare professionals. In many countries, such services are not readily available and thus could be seen as an added value in selection of the best-value biological product when offered by the supplier.^41^ However, an important requirement is that these services actually contribute to the value of the product. Furthermore, the value for particular services will strongly depend on the needs and expertise within the hospital, and the savings generated as a result of tenders could also be used to finance some of these services directly.^43^ On the other hand, considering additional services may be of particular interest in contexts where such services are not part of routine care.

### Environmental and sustainability factors.

When selecting a best-value biological medicine, part of that value also lies in the way in which the supplier has taken care of environmental aspects. The company’s policy on environmental factors such as production and transport could therefore be considered. Green Public Procurement therefore refers to environmental criteria in addition to traditional selection criteria such as the price, quality, and technical modalities of a product.^44^ This part of purchasing decisions has gained attention during recent years, in particular from national and international legislations promoting sustainable patterns of purchasing.

On a product level, differences may especially relate to the packaging material. Pharmaceutical packaging refers to the technology of protecting pharmaceutical products for distribution, storage, and usage.^45,46^ Ecologically friendly packaging includes packaging material emerging from natural sources (ie, proteins, starch), which do not cause harm to the environment. Green packaging materials will often include a specific eco-label.^46,47^ These labels can be used to evaluate whether a product contains eco-friendly packaging material, in other words, whether the packaging material is either recyclable or biodegradable.

## Patient-driven criteria

### Product user-friendliness.

Favorable patient-related features of the product add value to the medicine and should especially be considered for SC-administered biologicals. For such products, the patient is often responsible for injecting the medication and a more user-friendly injection device may lead to favorable clinical outcomes in terms of adherence.^48^ A patient-intuitive device may thus score higher than a standard prefilled syringe. Biological medicines for SC administration are generally available in 3 main types of device: prefilled syringes, prefilled pens, and electronic devices, which range from less to more automation and technical features.^48,49^ Product availability in different/improved administration devices may address patient needs with self-injection. For example, patients with rheumatic diseases may prefer ergonomically adapted self-injection systems that help overcome issues with dexterity.^48^eTable 1 provides an example of differences in injection device for a selection of biologicals (reference product and biosimilars).

In addition to injection system characteristics, injection pain can be a differentiating factor. Certain formulations of the same biological have proven to be less painful than others when they are injected or administered.^50,51^ Several factors may influence injection site pain such as excipients, needle size, pH, buffer capacity, and injection volume. Both a more user-friendly and a less painful injection can improve quality of care and contribute to better medication adherence.^50-53^

An essential condition is that the added value in terms of user-friendliness be proven in a clinical setting (ie, availability of data to substantiate this).^54^ For example, certain formulations of adalimumab products claim less painful injections.^51,53,55^ However, evidence about possible beneficial effects of citrate-free formulations is considered weak.^53^

### Patient support programs (PSPs).

PSPs are a subtype of VAS, with specific attention to patient support. Such services have the objective of helping patients manage their medication regimens and improve therapy adherence. This may be particularly relevant in chronic treatments, where the latter is more problematic.^56-58^ As with all VAS, their value depends strongly on the needs of the setting where the biological medicine is dispensed.

Examples of PSPs include injection device training, educational material for patients, and adherence programs. A specific example relevant to the US context is patient assistance programs. These are programs offered by suppliers by providing financial assistance to individual patients to partially cover their drug costs.^59^

## Assignment of relative weights and decision-making

Once the relevant criteria are identified, their relative weights, ie, the impact they have on the decision, need to be determined. The weights given to the criteria should be proportional to their respective relative importance. The relative weight that is assigned to each criterion is a subject for discussion and can vary across settings and countries.^14^ Although determination of weights is context dependent, sufficient weights need to be attributed to elements other than price for them to have an impact on decision-making.^13^

Inclusion and assignment of weights depends on the context of the product (class). For example, consideration of the product’s user-friendliness will only be relevant for SC products. The relevance of certain criteria and their weighting may also depend on the dispensing context. For instance, the importance assigned to VAS may vary across hospitals. Hospital pharmacies with limited capacity and biosimilar expertise to organize these services themselves may deem this important, whereas others may wish to organize them in house and allocate no or no significant weight to this in the decision. Third, the healthcare system decision-making context may have a role in attributing more weight to some factors than others.

As such, assignment of individual weights to the criteria requires a dynamic approach. Those undertaking product selection need to decide on relative weights for the selection criteria based on the context of the product and their hospital, making tailored but nonetheless transparent and evidence-based product selection decisions.

Criteria must be formulated as objectively measurable questions, to ensure objective and transparent assessment. In Box 1, the criteria are provided in question format. Answers need to be supported with data and/or other documents (eg, scientific publications, the European Public Assessment Report [EPAR], US prescribing information [USPI], production planning, history of recalls) to allow for an objective and evidence-based assessment.^6,7^ In addition to formulating criteria and determining their relative weights, how the answers will be scored needs to be prospectively defined (eg, 100% of score awarded if the answer falls under answer category A, 90% of score awarded if the answer falls under answer category B, etc).

Evaluating candidates to select the best-value biological medicine is product and context dependent. Hence, the provided guidance should be translated and tailored to each specific situation. Hospital pharmacists have a responsibility to apply this model to their particular local or regional context.

## Discussion

Although biosimilars and reference biologicals offer the same clinical outcomes, other criteria beyond price can be relevant in the decision-making process. Clinicians need to make informed decisions when selecting best-value biological medicine(s), and they need transparent and rational selection criteria to guide them during this process. This article provides an up-to-date overview on criteria that may be useful to consider and that may aid hospital pharmacists and clinicians with decision-making in practice. Because the context differs between products and the needs within regions or hospitals may vary, the provided guidance should be translated and tailored to each specific situation.^18,60,61^ In addition, this model can be useful for the selection of best-value biologicals by payers, health insurers, or pharmacy benefit managers in the US.

Evaluation of best-value biologicals based on the advanced model may facilitate transparent consideration of both price and qualitative criteria in decision-making. The proposed selection criteria in this article are categorized into product-driven, service-driven, and patient-driven criteria. Criteria in each of these categories may add value to the biological product and/or may impact practical implementation of the product.

The term “best-value biologicals” has been advanced to emphasize the focus on improving patient outcomes while maintaining an affordable medicine bill, rather than focusing on either originator or biosimilar uptake, as both contribute to a sustainable off-patent biologicals marketplace.^62^

National authorities have already been actively involved in guiding purchasers to select the best-value biological. In Ireland, the Health Service Executive established a best-value biologicals program in 2019 for the off-patent tumor necrosis factor (TNF)-α inhibitors etanercept and adalimumab.^63^ In this context, an exhaustive list of 13 criteria was formulated to select the best-value biologicals etanercept and adalimumab. These criteria included acquisition costs, as well as qualitative criteria such as therapeutic indications, formulation considerations, and patient factors. This example underlines the importance of a more inclusive approach when selecting a best-value biological, as a biological’s value goes beyond price considerations alone, and may be informative for hospital pharmacists and other stakeholders in their decision-making. Differentiation based on these criteria may also have tangible clinical and practical impacts, both on purchasers and patients (eg, more user-friendly injection devices).

Selecting the best-value biological requires adequate understanding by clinicians of the science behind the development and evaluation of biosimilars and how regulatory frameworks are tailored to the biosimilarity principle.^13,64^ Besides this, pharmacists should, as pharmaceutical product specialists, be well informed regarding the qualitative aspects that bring product value and may have an important impact on practical implementation of a product in clinical care. While purchasing biologicals solely on the basis of price may generate important short-term savings, this approach may overlook important product characteristics and lead to less sustainable practices in the longer term.^13,65^ Achieving the lowest price possible for biological medicines may lead to market impoverishment. Instead, competition on value-adding criteria should be stimulated. In this way, companies are incentivized to innovate on product features such as dosage, package size, administration route, formulation, and patient-friendly injection devices.^66,67^ Importantly, EPARs, USPIs, or other scientific/regulatory documents should serve as a reliable reference to substantiate the value of additional differentiating criteria.

It should be noted that the selection process can and may lead to several best-value biologicals, instead of only one. In settings where the market volume allows multiple winners, selection of multiple winners should be strived for, as by stimulating market plurality it benefits both the sustainability of the market and the availability of the biological medicine for the patient.^13,68^

## Conclusion

With a growing number of biosimilar products becoming available on the US and European markets, hospital pharmacists have a wide range of off-patent biological products from which to choose. This article advances a model to select best-value biologicals, taking into account additional qualitative criteria besides price. While the model may facilitate informed and transparent decision-making, the overview of criteria and the allocated weights need to be adapted to the local and product-specific context.

**Box 1. Overview of Possible Questions Relevant to the Selection of a Best-Value Biological, Besides Price Considerations**

**I. Product-driven criteria** ^a^

**1. Technical product features**

- Q1. Are there any differences in the number of strengths available compared to the other candidate(s)?
- Q2. Are there any differences in product administration compared to the other candidate(s) (eg, administration route, infusion speed, vial protectors, temperature-sensitive indicator, less frequent administrations)?
- Q3. Are there any differences in formulation (excipients, stabilizers, etc) compared to the other candidate(s)?
- Q4. Are there any differences in the product’s reconstitution compared to the other candidate(s) (eg, ready-to-use formulation, dissolution rate)?
- Q5. Are there any differences in storage conditions (including shelf life) compared to the other candidate(s)?
- Q6. Are there any differences in packaging or labeling of the product compared to the other candidate(s) (ie, easiness to read, barcoding per dose, products per dose packaging (optimal package size with regard to copayment), volume of packaging, documentation regarding adherence to FMD or DSCSA, etc)?

**2. Indications: authorization and reimbursement status**

- Q1. Are there any differences in registered indications compared to the other candidate(s)?
- Q2. Are all registered indications reimbursed?

**3. Real-world product experience**

- Q1. Are there real-world data to substantiate claims regarding patient experience, injection pain, etc?

**II. Service-driven criteria**

**4. Supply conditions**

- Q1. How does the supplier ensure supply?
- Q2. How does the supplier ensure and document that product integrity is maintained from the production site to administration to the patient (eg, storage and handling)?
- Q3. Does the supplier maintain adequate levels of reserve product in stock (metric: stock volume vs batch frequency)?

**5. Value-added services**

- Q1. Does the company offer services that improve patient care and adherence in the hospital environment or in support of delivery of the medicine at home (eg, training of healthcare professionals, nurse services at home, etc)?
- Q2. Does the company support the performance of antibody testing in patients?

**6. Environmental and sustainability factors**

- Q1. Does the supplier make use of ecologically friendly policies for production and transport?
- Q2. Does the company make use of ecologically friendly packaging material for its product (ie, biodegradable or recyclable material)?

**III. Patient-driven criteria**

**7. Product user-friendliness**

- Q1. Are there differences in device user friendliness compared to the other candidate(s) (eg, flexible vials, patient-intuitive device, etc)?
- Q2. Are there several injection devices available to choose between (ie, prefilled syringes, prefilled pens, and electronic devices)?
- Q3. Are there proven differences regardinginjection site pain compared to the other candidate(s)?

**8. Patient support programs**

- Q1. Does the company offer patient-oriented services such as injection device training, educational material for patients, or patient adherence programs?

Abbreviations: DSCSA, Drug Supply Chain and Security Act; FMD, Falsified Medicines Directive; PFP, prefilled pen; PFS, prefilled syringe.

^a^The relevance and corresponding weight of the abovementioned criteria or questions will depend on the product (class) and local context.

## Supplementary Material

zxac235_suppl_Supplementary_Appendix
