# Supplementary material for: How to select a best-value biological medicine? A practical model to support hospital pharmacists
Source: Am J Health Syst Pharm. 2022 Aug 25;79(22):2001–11. doi: 10.1093/ajhp/zxac235 (PMC9452170; doi:10.1093/ajhp/zxac235)
Supplement: zxac235_suppl_Supplementary_Appendix [file zxac235_suppl_supplementary_appendix.docx]

**eTable 1.** Overview of differences in administration device, strength and pharmaceutical form of a selection of EU-approved off-patent biologicals (reference product and biosimilars): examples of adalimumab, infliximab, trastuzumab. (Adapted from Kurki et al. Drugs. 2021)

| **Product** | **Administration device** | **Strength** | **Pharmaceutical form** | **Reference product** | **Biosimilars** | | |
| --- | --- | --- | --- | --- | --- | --- | --- |
| **Adalimumab** |  |  |  | **Humira** | **Amgevita, Halimatoz, Hefiya, Hyrimoz** | **Hulio, Imraldi, Idacio** | **Amsparity** |
|  | Vial | 40 mg (50 mg/ml) | SC | X |  | X | X |
|  | PFS | 20 mg (50 mg/ml) | SC |  | X |  | X |
|  | PFS | 20 mg (100 mg/ml) | SC | X |  |  |  |
|  | PFS | 40 mg (50 mg/ml) | SC | X | X | X | X |
|  | PFS | 40 mg (100 mg/ml) | SC | X |  |  |  |
|  | PFS | 80 mg (100 mg/ml) | SC | X |  |  |  |
|  | Pre-filled pen | 40 mg (50 mg/ml) | SC | X | X | X | X |
|  | Pre-filled pen | 40 mg (100 mg/ml) | SC | X |  |  |  |
|  | Pre-filled pen | 80 mg (100 mg/ml) | SC | X |  |  |  |
| **Infliximab** |  |  |  | **Remicade** | **Remsima** | **Flixabi, Inflectra, Zessly** | |
|  | Vial | 100 mg (100mg/ml) | IV | X | X | X | |
|  | PFS | 120 mg (120mg/ml) | SC |  | X |  | |
|  | Pre-filled pen | 120 mg (120mg/ml) | SC |  | X |  | |
| **Trastuzumab** |  |  |  | **Herceptin** | **Zercepac** | **Herzuma, Kanjinti, Ogivri, Ontruzant, Trazimera** | |
|  | Vial | 150 mg (21 mg/ml) | IV | X | X | X | |
|  | Vial | 420 mg (21 mg/ml) | IV |  |  | X | |
|  | Vial | 600 mg (30 mg/ml) | SC | X |  |  | |
| Abbreviations: PFS, pre-filled syringe; IV, intravenous; SC, subcutaneous. | | | | | | | |
